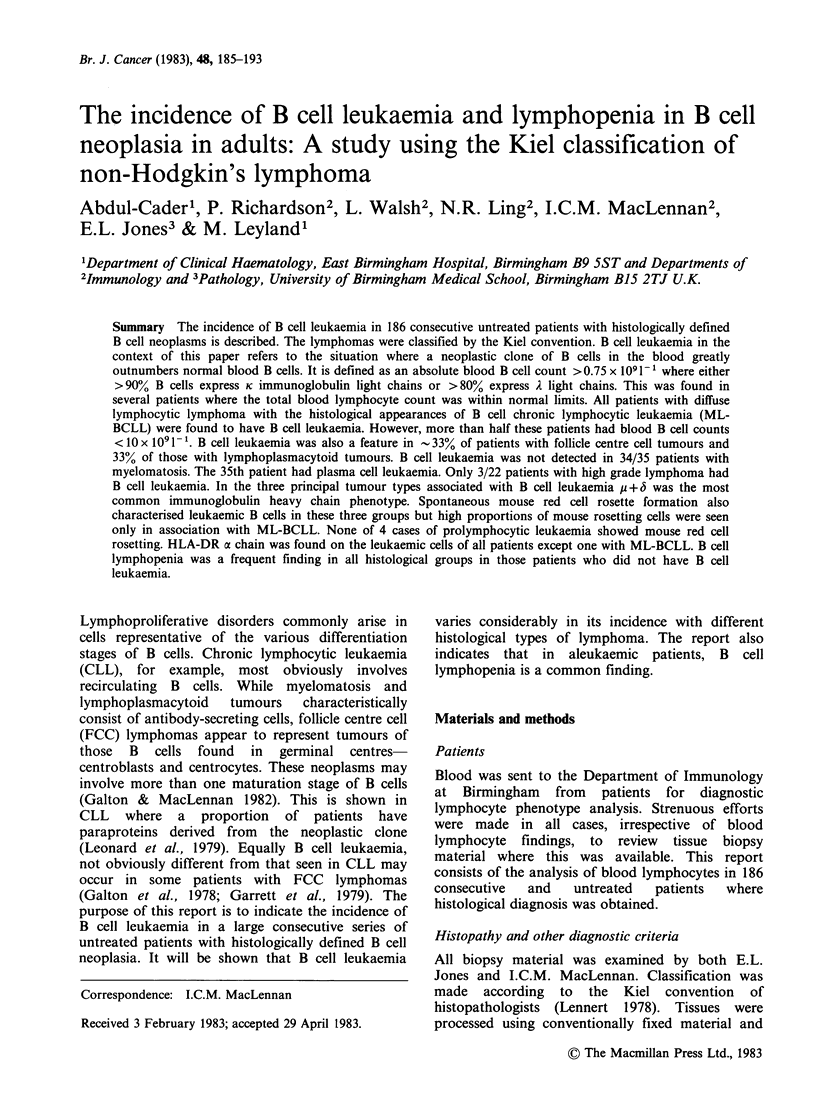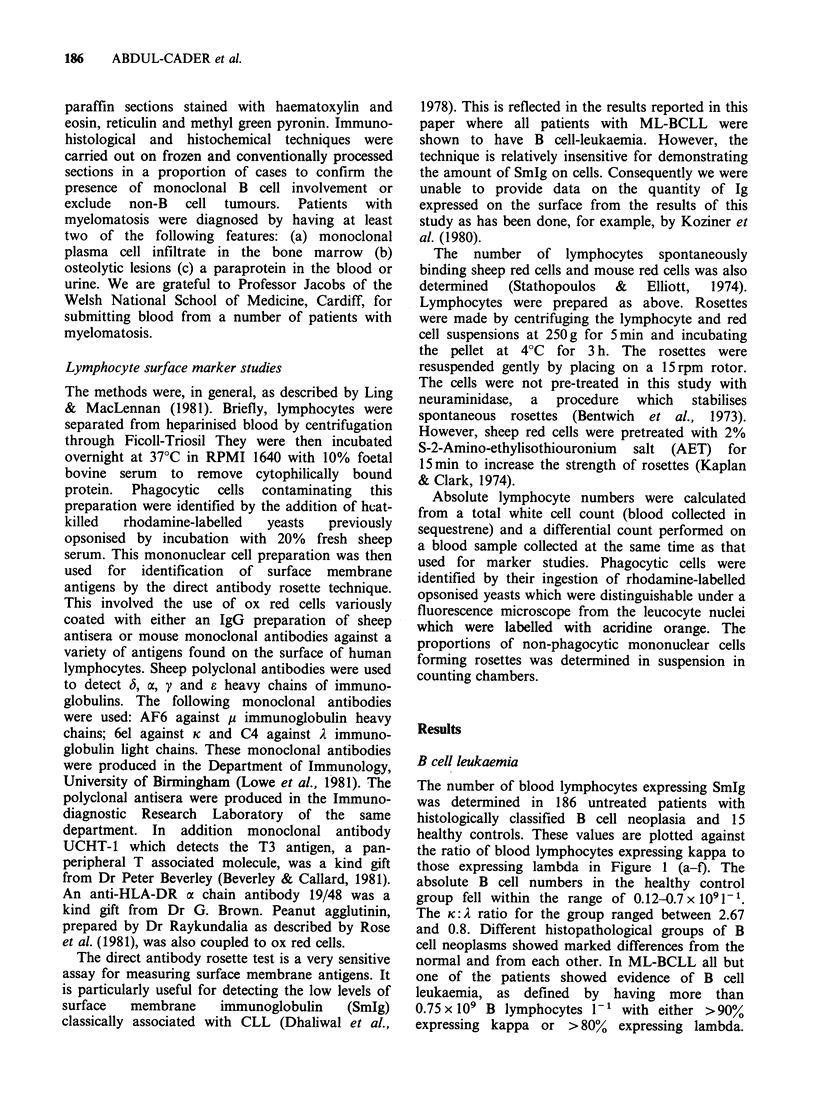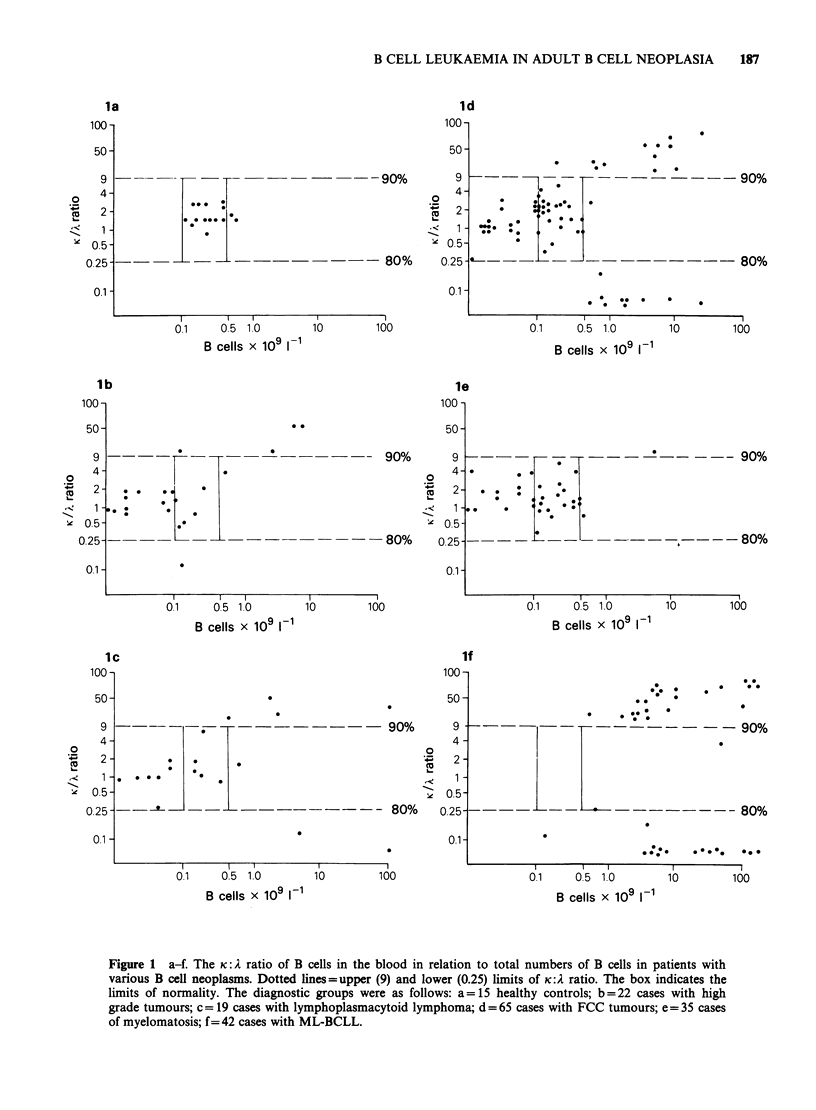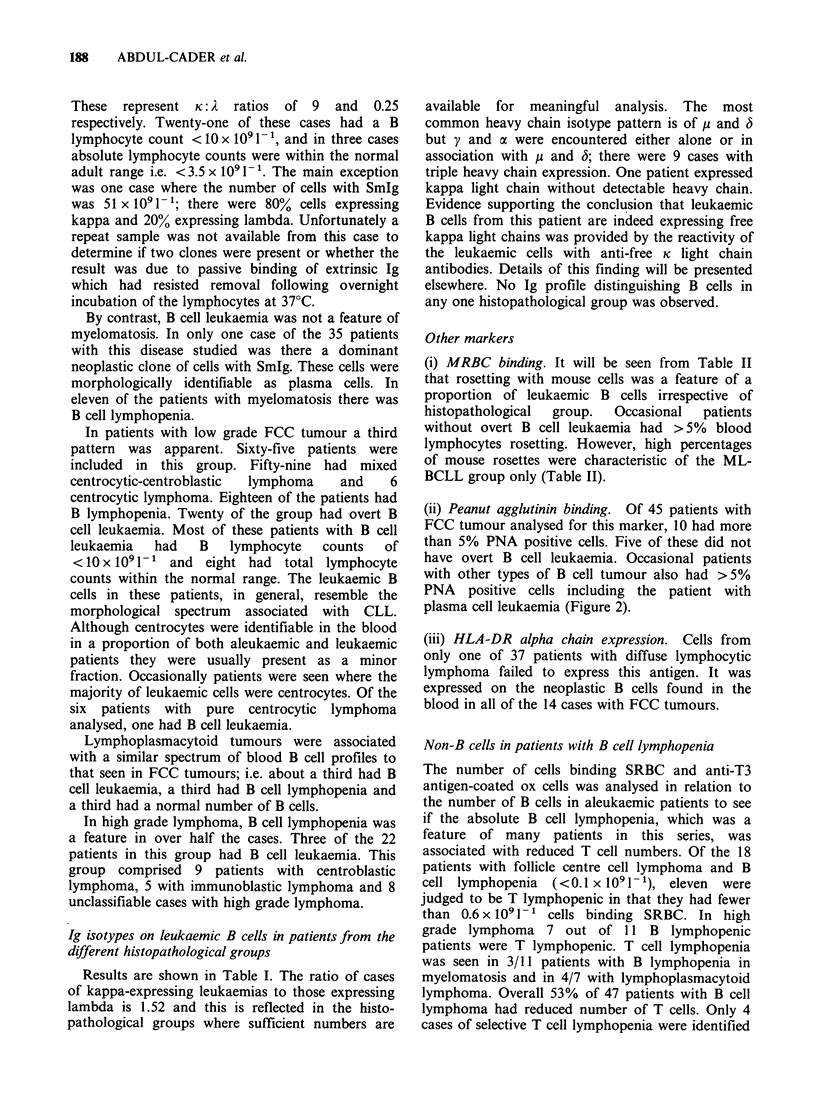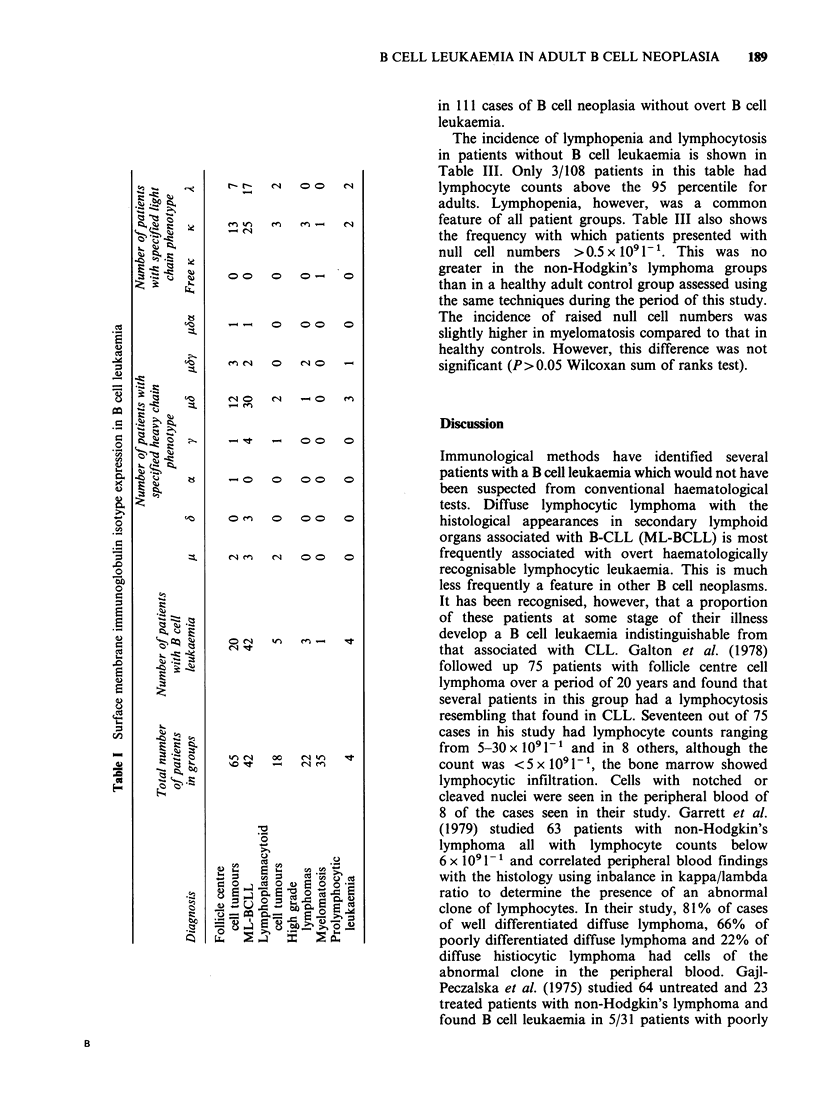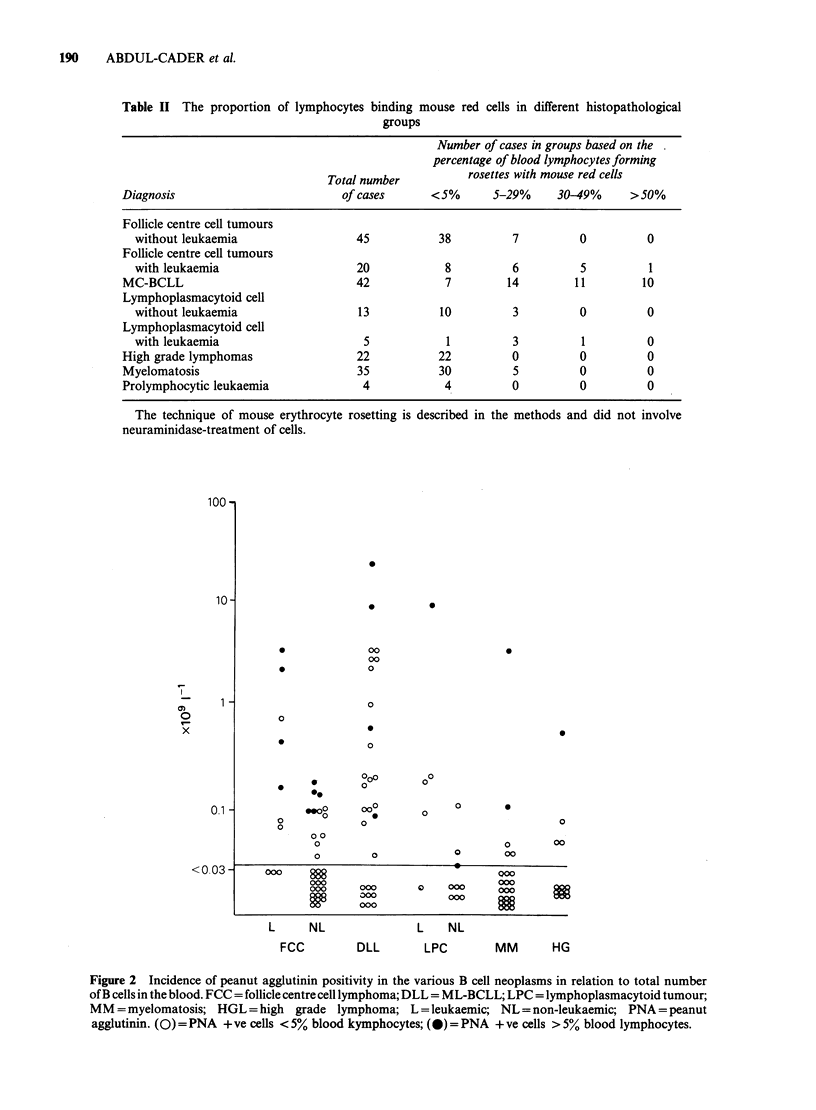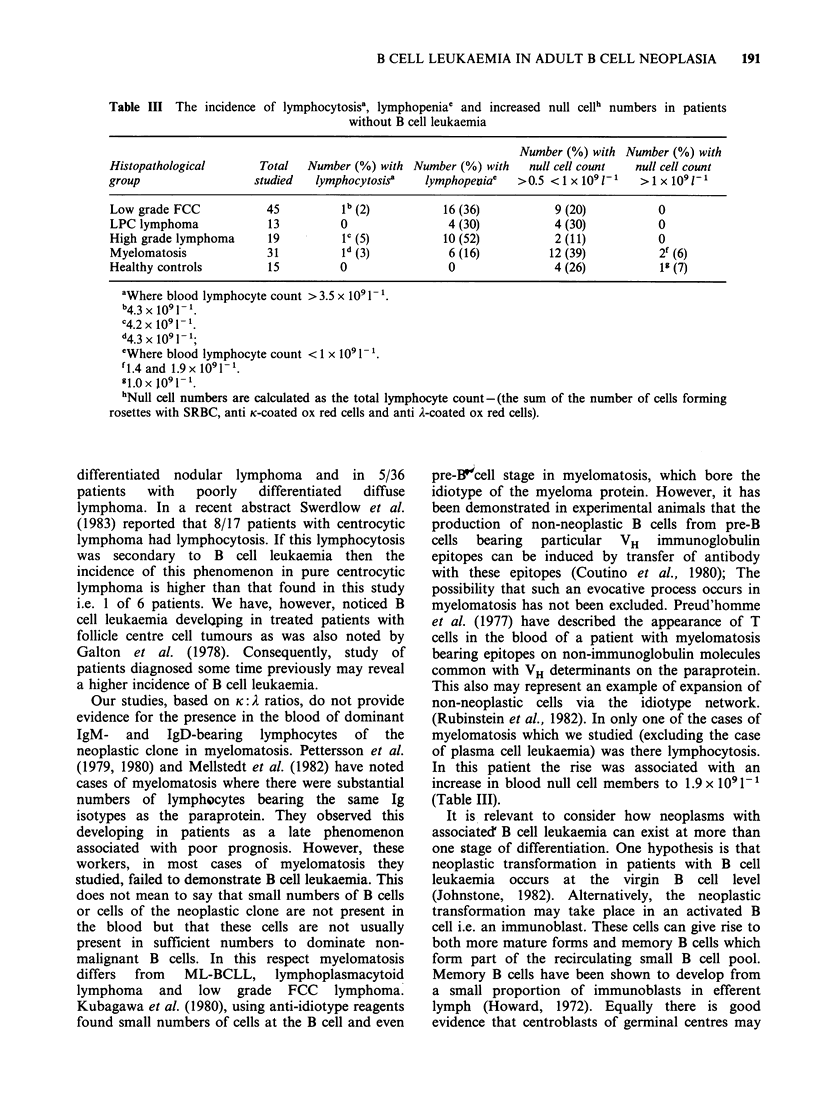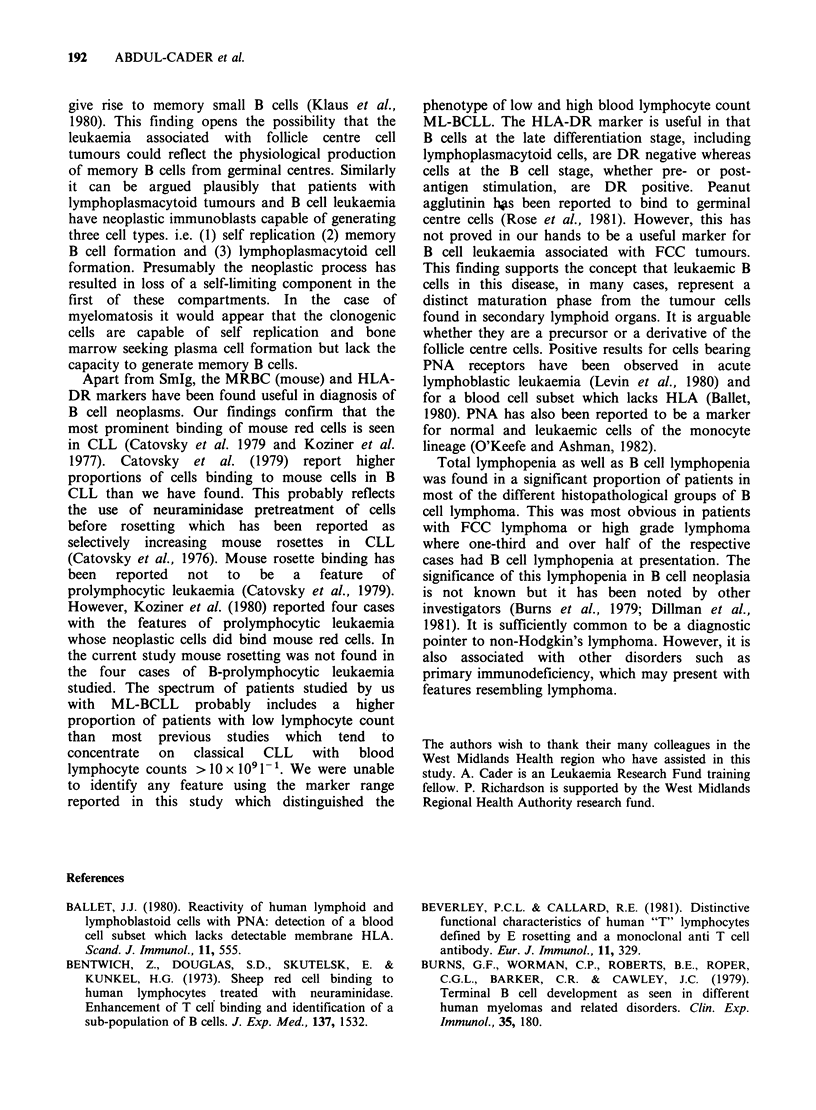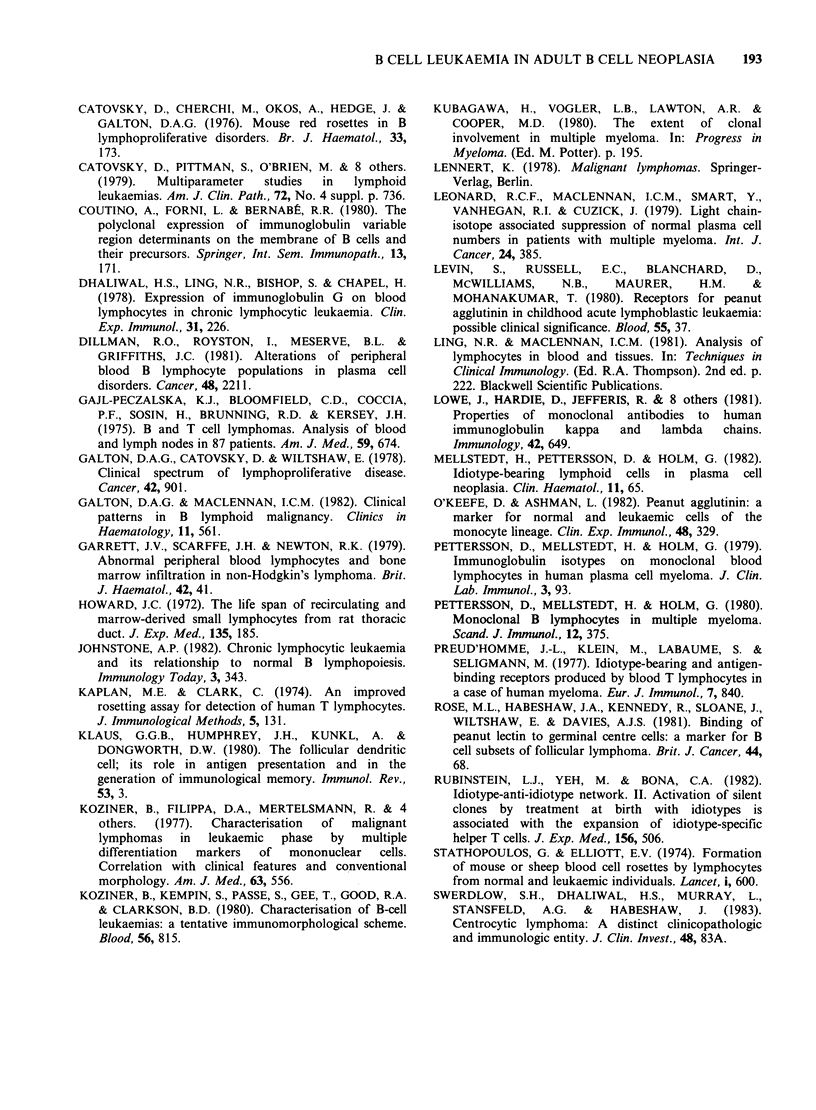# The incidence of B cell leukaemia and lymphopenia in B cell neoplasia in adults: a study using the Kiel classification of non-Hodgkin's lymphoma.

**DOI:** 10.1038/bjc.1983.174

**Published:** 1983-08

**Authors:** A. Cader, P. Richardson, L. Walsh, N. R. Ling, I. C. MacLennan, E. L. Jones, M. Leyland

## Abstract

The incidence of B cell leukaemia in 186 consecutive untreated patients with histologically defined B cell neoplasms is described. The lymphomas were classified by the Kiel convention. B cell leukaemia in the context of this paper refers to the situation where a neoplastic clone of B cells in the blood greatly outnumbers normal blood B cells. It is defined as an absolute blood B cell count greater than 0.75 X 10(9)1(-1) where either greater than 90% B cells express kappa immunoglobulin light chains or greater than 80% express lambda light chains. This was found in several patients where the total blood lymphocyte count was within normal limits. All patients with diffuse lymphocytic lymphoma with the histological appearances of B cell chronic lymphocytic leukaemia (ML-BCLL) were found to have B cell leukaemia. However, more than half these patients had blood B cell counts less than 10 X 10(9)1(-1). B cell leukaemia was also a feature in approximately 33% of patients with follicle centre cell tumours and 33% of those with lymphoplasmacytoid tumours. B cell leukaemia was not detected in 34/35 patients with myelomatosis. The 35th patient had plasma cell leukaemia. Only 3/22 patients with high grade lymphoma had B cell leukaemia. In the three principal tumour types associated with B cell leukaemia mu + delta was the most common immunoglobulin heavy chain phenotype. Spontaneous mouse red cell rosette formation also characterised leukaemic B cells in these three groups but high proportions of mouse rosetting cells were seen only in association with ML-BCLL. None of 4 cases of prolymphocytic leukaemia showed mouse red cell rosetting. HLA-DR alpha chain was found on the leukaemic cells of all patients except one with ML-BCLL. B cell lymphopenia was a frequent finding in all histological groups in those patients who did not have B cell leukaemia.


*Br. J. Cancer* (1983), **48**, 185–193

### The incidence of B cell leukaemia and lymphopenia in B cell neoplasia in adults: A study using the Kiel classification of non-Hodgkin's lymphoma

Abdul-Cader[1], P. Richardson[2], L. Walsh[2], N.R. Ling[2], I.C.M. MacLennan[2], E.L. Jones[3] & M. Leyland[1]

[1]*Department of Clinical Haematology, East Birmingham Hospital, Birmingham B9 5ST and Departments of* [2]*Immunology and* [3]*Pathology, University of Birmingham Medical School, Birmingham B15 2TJ U.K.*

**Summary** The incidence of B cell leukaemia in 186 consecutive untreated patients with histologically defined B cell neoplasms is described. The lymphomas were classified by the Kiel convention. B cell leukaemia in the context of this paper refers to the situation where a neoplastic clone of B cells in the blood greatly outnumbers normal blood B cells. It is defined as an absolute blood B cell count $>0.75 \times 10^9 l^{-1}$ where either >90% B cells express $\kappa$ immunoglobulin light chains or >80% express $\lambda$ light chains. This was found in several patients where the total blood lymphocyte count was within normal limits. All patients with diffuse lymphocytic lymphoma with the histological appearances of B cell chronic lymphocytic leukaemia (ML-BCLL) were found to have B cell leukaemia. However, more than half these patients had blood B cell counts $<10 \times 10^9 l^{-1}$. B cell leukaemia was also a feature in ~33% of patients with follicle centre cell tumours and 33% of those with lymphoplasmacytoid tumours. B cell leukaemia was not detected in 34/35 patients with myelomatosis. The 35th patient had plasma cell leukaemia. Only 3/22 patients with high grade lymphoma had B cell leukaemia. In the three principal tumour types associated with B cell leukaemia $\mu+\delta$ was the most common immunoglobulin heavy chain phenotype. Spontaneous mouse red cell rosette formation also characterised leukaemic B cells in these three groups but high proportions of mouse rosetting cells were seen only in association with ML-BCLL. None of 4 cases of prolymphocytic leukaemia showed mouse red cell rosetting. HLA-DR $\alpha$ chain was found on the leukaemic cells of all patients except one with ML-BCLL. B cell lymphopenia was a frequent finding in all histological groups in those patients who did not have B cell leukaemia.

Lymphoproliferative disorders commonly arise in cells representative of the various differentiation stages of B cells. Chronic lymphocytic leukaemia (CLL), for example, most obviously involves recirculating B cells. While myelomatosis and lymphoplasmacytoid tumours characteristically consist of antibody-secreting cells, follicle centre cell (FCC) lymphomas appear to represent tumours of those B cells found in germinal centres—centroblasts and centrocytes. These neoplasms may involve more than one maturation stage of B cells (Galton & MacLennan 1982). This is shown in CLL where a proportion of patients have paraproteins derived from the neoplastic clone (Leonard *et al.*, 1979). Equally B cell leukaemia, not obviously different from that seen in CLL may occur in some patients with FCC lymphomas (Galton *et al.*, 1978; Garrett *et al.*, 1979). The purpose of this report is to indicate the incidence of B cell leukaemia in a large consecutive series of untreated patients with histologically defined B cell neoplasia. It will be shown that B cell leukaemia varies considerably in its incidence with different histological types of lymphoma. The report also indicates that in aleukaemic patients, B cell lymphopenia is a common finding.

Correspondence: I.C.M. MacLennan

Received 3 February 1983; accepted 29 April 1983.

### Materials and methods

### *Patients*

Blood was sent to the Department of Immunology at Birmingham from patients for diagnostic lymphocyte phenotype analysis. Strenuous efforts were made in all cases, irrespective of blood lymphocyte findings, to review tissue biopsy material where this was available. This report consists of the analysis of blood lymphocytes in 186 consecutive and untreated patients where histological diagnosis was obtained.

### *Histopathy and other diagnostic criteria*

All biopsy material was examined by both E.L. Jones and I.C.M. MacLennan. Classification was made according to the Kiel convention of histopathologists (Lennert 1978). Tissues were processed using conventionally fixed material and

© The Macmillan Press Ltd., 1983

paraffin sections stained with haematoxylin and eosin, reticulin and methyl green pyronin. Immunohistological and histochemical techniques were carried out on frozen and conventionally processed sections in a proportion of cases to confirm the presence of monoclonal B cell involvement or exclude non-B cell tumours. Patients with myelomatosis were diagnosed by having at least two of the following features: (a) monoclonal plasma cell infiltrate in the bone marrow (b) osteolytic lesions (c) a paraprotein in the blood or urine. We are grateful to Professor Jacobs of the Welsh National School of Medicine, Cardiff, for submitting blood from a number of patients with myelomatosis.

*Lymphocyte surface marker studies*

The methods were, in general, as described by Ling & MacLennan (1981). Briefly, lymphocytes were separated from heparinised blood by centrifugation through Ficoll-Triosil They were then incubated overnight at 37°C in RPMI 1640 with 10% foetal bovine serum to remove cytophilically bound protein. Phagocytic cells contaminating this preparation were identified by the addition of heat-killed rhodamine-labelled yeasts previously opsonised by incubation with 20% fresh sheep serum. This mononuclear cell preparation was then used for identification of surface membrane antigens by the direct antibody rosette technique. This involved the use of ox red cells variously coated with either an IgG preparation of sheep antisera or mouse monoclonal antibodies against a variety of antigens found on the surface of human lymphocytes. Sheep polyclonal antibodies were used to detect $\delta$, $\alpha$, $\gamma$ and $\varepsilon$ heavy chains of immunoglobulins. The following monoclonal antibodies were used: AF6 against $\mu$ immunoglobulin heavy chains; 6el against $\kappa$ and C4 against $\lambda$ immunoglobulin light chains. These monoclonal antibodies were produced in the Department of Immunology, University of Birmingham (Lowe *et al.*, 1981). The polyclonal antisera were produced in the Immunodiagnostic Research Laboratory of the same department. In addition monoclonal antibody UCHT-1 which detects the T3 antigen, a pan-peripheral T associated molecule, was a kind gift from Dr Peter Beverley (Beverley & Callard, 1981). An anti-HLA-DR $\alpha$ chain antibody 19/48 was a kind gift from Dr G. Brown. Peanut agglutinin, prepared by Dr Raykundalia as described by Rose *et al.* (1981), was also coupled to ox red cells.

The direct antibody rosette test is a very sensitive assay for measuring surface membrane antigens. It is particularly useful for detecting the low levels of surface membrane immunoglobulin (SmIg) classically associated with CLL (Dhaliwal *et al.*, 1978). This is reflected in the results reported in this paper where all patients with ML-BCLL were shown to have B cell-leukaemia. However, the technique is relatively insensitive for demonstrating the amount of SmIg on cells. Consequently we were unable to provide data on the quantity of Ig expressed on the surface from the results of this study as has been done, for example, by Koziner *et al.* (1980).

The number of lymphocytes spontaneously binding sheep red cells and mouse red cells was also determined (Stathopoulos & Elliott, 1974). Lymphocytes were prepared as above. Rosettes were made by centrifuging the lymphocyte and red cell suspensions at 250 g for 5 min and incubating the pellet at 4°C for 3 h. The rosettes were resuspended gently by placing on a 15 rpm rotor. The cells were not pre-treated in this study with neuraminidase, a procedure which stabilises spontaneous rosettes (Bentwich *et al.*, 1973). However, sheep red cells were pretreated with 2% S-2-Amino-ethylisothiouronium salt (AET) for 15 min to increase the strength of rosettes (Kaplan & Clark, 1974).

Absolute lymphocyte numbers were calculated from a total white cell count (blood collected in sequestrene) and a differential count performed on a blood sample collected at the same time as that used for marker studies. Phagocytic cells were identified by their ingestion of rhodamine-labelled opsonised yeasts which were distinguishable under a fluorescence microscope from the leucocyte nuclei which were labelled with acridine orange. The proportions of non-phagocytic mononuclear cells forming rosettes was determined in suspension in counting chambers.

### Results

*B cell leukaemia*

The number of blood lymphocytes expressing SmIg was determined in 186 untreated patients with histologically classified B cell neoplasia and 15 healthy controls. These values are plotted against the ratio of blood lymphocytes expressing kappa to those expressing lambda in Figure 1 (a–f). The absolute B cell numbers in the healthy control group fell within the range of $0.12–0.7 \times 10^9 l^{-1}$. The $\kappa:\lambda$ ratio for the group ranged between 2.67 and 0.8. Different histopathological groups of B cell neoplasms showed marked differences from the normal and from each other. In ML-BCLL all but one of the patients showed evidence of B cell leukaemia, as defined by having more than $0.75 \times 10^9$ B lymphocytes $l^{-1}$ with either >90% expressing kappa or >80% expressing lambda.

**Figure 1** a–f. The $\kappa:\lambda$ ratio of B cells in the blood in relation to total numbers of B cells in patients with various B cell neoplasms. Dotted lines = upper (9) and lower (0.25) limits of $\kappa:\lambda$ ratio. The box indicates the limits of normality. The diagnostic groups were as follows: a = 15 healthy controls; b = 22 cases with high grade tumours; c = 19 cases with lymphoplasmacytoid lymphoma; d = 65 cases with FCC tumours; e = 35 cases of myelomatosis; f = 42 cases with ML-BCLL.

These represent $\kappa:\lambda$ ratios of 9 and 0.25 respectively. Twenty-one of these cases had a B lymphocyte count $<10 \times 10^9 l^{-1}$, and in three cases absolute lymphocyte counts were within the normal adult range i.e. $<3.5 \times 10^9 l^{-1}$. The main exception was one case where the number of cells with SmIg was $51 \times 10^9 l^{-1}$; there were 80% cells expressing kappa and 20% expressing lambda. Unfortunately a repeat sample was not available from this case to determine if two clones were present or whether the result was due to passive binding of extrinsic Ig which had resisted removal following overnight incubation of the lymphocytes at 37°C.

By contrast, B cell leukaemia was not a feature of myelomatosis. In only one case of the 35 patients with this disease studied was there a dominant neoplastic clone of cells with SmIg. These cells were morphologically identifiable as plasma cells. In eleven of the patients with myelomatosis there was B cell lymphopenia.

In patients with low grade FCC tumour a third pattern was apparent. Sixty-five patients were included in this group. Fifty-nine had mixed centrocytic-centroblastic lymphoma and 6 centrocytic lymphoma. Eighteen of the patients had B lymphopenia. Twenty of the group had overt B cell leukaemia. Most of these patients with B cell leukaemia had B lymphocyte counts of $<10 \times 10^9 l^{-1}$ and eight had total lymphocyte counts within the normal range. The leukaemic B cells in these patients, in general, resemble the morphological spectrum associated with CLL. Although centrocytes were identifiable in the blood in a proportion of both aleukaemic and leukaemic patients they were usually present as a minor fraction. Occasionally patients were seen where the majority of leukaemic cells were centrocytes. Of the six patients with pure centrocytic lymphoma analysed, one had B cell leukaemia.

Lymphoplasmacytoid tumours were associated with a similar spectrum of blood B cell profiles to that seen in FCC tumours; i.e. about a third had B cell leukaemia, a third had B cell lymphopenia and a third had a normal number of B cells.

In high grade lymphoma, B cell lymphopenia was a feature in over half the cases. Three of the 22 patients in this group had B cell leukaemia. This group comprised 9 patients with centroblastic lymphoma, 5 with immunoblastic lymphoma and 8 unclassifiable cases with high grade lymphoma.

*Ig isotypes on leukaemic B cells in patients from the different histopathological groups*

Results are shown in Table I. The ratio of cases of kappa-expressing leukaemias to those expressing lambda is 1.52 and this is reflected in the histopathological groups where sufficient numbers are available for meaningful analysis. The most common heavy chain isotype pattern is of $\mu$ and $\delta$ but $\gamma$ and $\alpha$ were encountered either alone or in association with $\mu$ and $\delta$; there were 9 cases with triple heavy chain expression. One patient expressed kappa light chain without detectable heavy chain. Evidence supporting the conclusion that leukaemic B cells from this patient are indeed expressing free kappa light chains was provided by the reactivity of the leukaemic cells with anti-free $\kappa$ light chain antibodies. Details of this finding will be presented elsewhere. No Ig profile distinguishing B cells in any one histopathological group was observed.

*Other markers*

(i) *MRBC binding*. It will be seen from Table II that rosetting with mouse cells was a feature of a proportion of leukaemic B cells irrespective of histopathological group. Occasional patients without overt B cell leukaemia had >5% blood lymphocytes rosetting. However, high percentages of mouse rosettes were characteristic of the ML-BCLL group only (Table II).

(ii) *Peanut agglutinin binding*. Of 45 patients with FCC tumour analysed for this marker, 10 had more than 5% PNA positive cells. Five of these did not have overt B cell leukaemia. Occasional patients with other types of B cell tumour also had >5% PNA positive cells including the patient with plasma cell leukaemia (Figure 2).

(iii) *HLA-DR alpha chain expression*. Cells from only one of 37 patients with diffuse lymphocytic lymphoma failed to express this antigen. It was expressed on the neoplastic B cells found in the blood in all of the 14 cases with FCC tumours.

*Non-B cells in patients with B cell lymphopenia*

The number of cells binding SRBC and anti-T3 antigen-coated ox cells was analysed in relation to the number of B cells in aleukaemic patients to see if the absolute B cell lymphopenia, which was a feature of many patients in this series, was associated with reduced T cell numbers. Of the 18 patients with follicle centre cell lymphoma and B cell lymphopenia ($<0.1 \times 10^9 l^{-1}$), eleven were judged to be T lymphopenic in that they had fewer than $0.6 \times 10^9 l^{-1}$ cells binding SRBC. In high grade lymphoma 7 out of 11 B lymphopenic patients were T lymphopenic. T cell lymphopenia was seen in 3/11 patients with B lymphopenia in myelomatosis and in 4/7 with lymphoplasmacytoid lymphoma. Overall 53% of 47 patients with B cell lymphoma had reduced number of T cells. Only 4 cases of selective T cell lymphopenia were identified

**Table I** Surface membrane immunoglobulin isotype expression in B cell leukaemia

| *Diagnosis* | *Total number of patients in groups* | *Number of patients with B cell leukaemia* | *Number of patients with specified heavy chain phenotype* | | | | | | | *Number of patients with specified light chain phenotype* | | |
|---|---|---|---|---|---|---|---|---|---|---|---|---|
| | | | *μ* | *δ* | *α* | *γ* | *μδ* | *μδγ* | *μδα* | *Free κ* | *κ* | *λ* |
| Follicle centre cell tumours | 65 | 20 | 2 | 0 | 1 | 1 | 12 | 3 | 1 | 0 | 13 | 7 |
| ML-BCLL | 42 | 42 | 3 | 3 | 0 | 4 | 30 | 2 | 1 | 0 | 25 | 17 |
| Lymphoplasmacytoid cell tumours | 18 | 5 | 2 | 0 | 0 | 1 | 2 | 0 | 0 | 0 | 3 | 2 |
| High grade lymphomas | 22 | 3 | 0 | 0 | 0 | 0 | 1 | 2 | 0 | 0 | 3 | 0 |
| Myelomatosis | 35 | 1 | 0 | 0 | 0 | 0 | 0 | 0 | 0 | 1 | 1 | 0 |
| Prolymphocytic leukaemia | 4 | 4 | 0 | 0 | 0 | 0 | 3 | 1 | 0 | 0 | 2 | 2 |

in 111 cases of B cell neoplasia without overt B cell leukaemia.

The incidence of lymphopenia and lymphocytosis in patients without B cell leukaemia is shown in Table III. Only 3/108 patients in this table had lymphocyte counts above the 95 percentile for adults. Lymphopenia, however, was a common feature of all patient groups. Table III also shows the frequency with which patients presented with null cell numbers $>0.5\times10^{9}\,l^{-1}$. This was no greater in the non-Hodgkin's lymphoma groups than in a healthy adult control group assessed using the same techniques during the period of this study. The incidence of raised null cell numbers was slightly higher in myelomatosis compared to that in healthy controls. However, this difference was not significant ($P>0.05$ Wilcoxan sum of ranks test).

### Discussion

Immunological methods have identified several patients with a B cell leukaemia which would not have been suspected from conventional haematological tests. Diffuse lymphocytic lymphoma with the histological appearances in secondary lymphoid organs associated with B-CLL (ML-BCLL) is most frequently associated with overt haematologically recognisable lymphocytic leukaemia. This is much less frequently a feature in other B cell neoplasms. It has been recognised, however, that a proportion of these patients at some stage of their illness develop a B cell leukaemia indistinguishable from that associated with CLL. Galton *et al.* (1978) followed up 75 patients with follicle centre cell lymphoma over a period of 20 years and found that several patients in this group had a lymphocytosis resembling that found in CLL. Seventeen out of 75 cases in his study had lymphocyte counts ranging from $5–30\times10^{9}\,l^{-1}$ and in 8 others, although the count was $<5\times10^{9}\,l^{-1}$, the bone marrow showed lymphocytic infiltration. Cells with notched or cleaved nuclei were seen in the peripheral blood of 8 of the cases seen in their study. Garrett *et al.* (1979) studied 63 patients with non-Hodgkin's lymphoma all with lymphocyte counts below $6\times10^{9}\,l^{-1}$ and correlated peripheral blood findings with the histology using inbalance in kappa/lambda ratio to determine the presence of an abnormal clone of lymphocytes. In their study, 81% of cases of well differentiated diffuse lymphoma, 66% of poorly differentiated diffuse lymphoma and 22% of diffuse histiocytic lymphoma had cells of the abnormal clone in the peripheral blood. Gajl-Peczalska *et al.* (1975) studied 64 untreated and 23 treated patients with non-Hodgkin's lymphoma and found B cell leukaemia in 5/31 patients with poorly

**Table II** The proportion of lymphocytes binding mouse red cells in different histopathological groups

| Diagnosis | Total number of cases | Number of cases in groups based on the percentage of blood lymphocytes forming rosettes with mouse red cells <5% | 5–29% | 30–49% | >50% |
|---|---|---|---|---|---|
| Follicle centre cell tumours without leukaemia | 45 | 38 | 7 | 0 | 0 |
| Follicle centre cell tumours with leukaemia | 20 | 8 | 6 | 5 | 1 |
| MC-BCLL | 42 | 7 | 14 | 11 | 10 |
| Lymphoplasmacytoid cell without leukaemia | 13 | 10 | 3 | 0 | 0 |
| Lymphoplasmacytoid cell with leukaemia | 5 | 1 | 3 | 1 | 0 |
| High grade lymphomas | 22 | 22 | 0 | 0 | 0 |
| Myelomatosis | 35 | 30 | 5 | 0 | 0 |
| Prolymphocytic leukaemia | 4 | 4 | 0 | 0 | 0 |

The technique of mouse erythrocyte rosetting is described in the methods and did not involve neuraminidase-treatment of cells.

**Figure 2** Incidence of peanut agglutinin positivity in the various B cell neoplasms in relation to total number of B cells in the blood. FCC = follicle centre cell lymphoma; DLL = ML-BCLL; LPC = lymphoplasmacytoid tumour; MM = myelomatosis; HGL = high grade lymphoma; L = leukaemic; NL = non-leukaemic; PNA = peanut agglutinin. (○) = PNA +ve cells <5% blood kymphocytes; (●) = PNA +ve cells >5% blood lymphocytes.

**Table III** The incidence of lymphocytosis[a], lymphopenia[e] and increased null cell[h] numbers in patients without B cell leukaemia

| *Histopathological group* | *Total studied* | *Number (%) with lymphocytosis*[a] | *Number (%) with lymphopenia*[e] | *Number (%) with null cell count* $>0.5 <1\times10^9 l^{-1}$ | *Number (%) with null cell count* $>1\times10^9 l^{-1}$ |
|---|---|---|---|---|---|
| Low grade FCC | 45 | 1[b] (2) | 16 (36) | 9 (20) | 0 |
| LPC lymphoma | 13 | 0 | 4 (30) | 4 (30) | 0 |
| High grade lymphoma | 19 | 1[c] (5) | 10 (52) | 2 (11) | 0 |
| Myelomatosis | 31 | 1[d] (3) | 6 (16) | 12 (39) | 2[f] (6) |
| Healthy controls | 15 | 0 | 0 | 4 (26) | 1[g] (7) |

[a]Where blood lymphocyte count $>3.5\times10^9 l^{-1}$.

[b]$4.3\times10^9 l^{-1}$.

[c]$4.2\times10^9 l^{-1}$.

[d]$4.3\times10^9 l^{-1}$;

[e]Where blood lymphocyte count $<1\times10^9 l^{-1}$.

[f]1.4 and $1.9\times10^9 l^{-1}$.

[g]$1.0\times10^9 l^{-1}$.

[h]Null cell numbers are calculated as the total lymphocyte count—(the sum of the number of cells forming rosettes with SRBC, anti $\kappa$-coated ox red cells and anti $\lambda$-coated ox red cells).

differentiated nodular lymphoma and in 5/36 patients with poorly differentiated diffuse lymphoma. In a recent abstract Swerdlow *et al.* (1983) reported that 8/17 patients with centrocytic lymphoma had lymphocytosis. If this lymphocytosis was secondary to B cell leukaemia then the incidence of this phenomenon in pure centrocytic lymphoma is higher than that found in this study i.e. 1 of 6 patients. We have, however, noticed B cell leukaemia developing in treated patients with follicle centre cell tumours as was also noted by Galton *et al.* (1978). Consequently, study of patients diagnosed some time previously may reveal a higher incidence of B cell leukaemia.

Our studies, based on $\kappa:\lambda$ ratios, do not provide evidence for the presence in the blood of dominant IgM- and IgD-bearing lymphocytes of the neoplastic clone in myelomatosis. Pettersson *et al.* (1979, 1980) and Mellstedt *et al.* (1982) have noted cases of myelomatosis where there were substantial numbers of lymphocytes bearing the same Ig isotypes as the paraprotein. They observed this developing in patients as a late phenomenon associated with poor prognosis. However, these workers, in most cases of myelomatosis they studied, failed to demonstrate B cell leukaemia. This does not mean to say that small numbers of B cells or cells of the neoplastic clone are not present in the blood but that these cells are not usually present in sufficient numbers to dominate non-malignant B cells. In this respect myelomatosis differs from ML-BCLL, lymphoplasmacytoid lymphoma and low grade FCC lymphoma. Kubagawa *et al.* (1980), using anti-idiotype reagents found small numbers of cells at the B cell and even pre-B cell stage in myelomatosis, which bore the idiotype of the myeloma protein. However, it has been demonstrated in experimental animals that the production of non-neoplastic B cells from pre-B cells bearing particular $V_H$ immunoglobulin epitopes can be induced by transfer of antibody with these epitopes (Coutino *et al.*, 1980); The possibility that such an evocative process occurs in myelomatosis has not been excluded. Preud'homme *et al.* (1977) have described the appearance of T cells in the blood of a patient with myelomatosis bearing epitopes on non-immunoglobulin molecules common with $V_H$ determinants on the paraprotein. This also may represent an example of expansion of non-neoplastic cells via the idiotype network. (Rubinstein *et al.*, 1982). In only one of the cases of myelomatosis which we studied (excluding the case of plasma cell leukaemia) was there lymphocytosis. In this patient the rise was associated with an increase in blood null cell members to $1.9\times10^9 l^{-1}$ (Table III).

It is relevant to consider how neoplasms with associated B cell leukaemia can exist at more than one stage of differentiation. One hypothesis is that neoplastic transformation in patients with B cell leukaemia occurs at the virgin B cell level (Johnstone, 1982). Alternatively, the neoplastic transformation may take place in an activated B cell i.e. an immunoblast. These cells can give rise to both more mature forms and memory B cells which form part of the recirculating small B cell pool. Memory B cells have been shown to develop from a small proportion of immunoblasts in efferent lymph (Howard, 1972). Equally there is good evidence that centroblasts of germinal centres may give rise to memory small B cells (Klaus *et al.*, 1980). This finding opens the possibility that the leukaemia associated with follicle centre cell tumours could reflect the physiological production of memory B cells from germinal centres. Similarly it can be argued plausibly that patients with lymphoplasmacytoid tumours and B cell leukaemia have neoplastic immunoblasts capable of generating three cell types. i.e. (1) self replication (2) memory B cell formation and (3) lymphoplasmacytoid cell formation. Presumably the neoplastic process has resulted in loss of a self-limiting component in the first of these compartments. In the case of myelomatosis it would appear that the clonogenic cells are capable of self replication and bone marrow seeking plasma cell formation but lack the capacity to generate memory B cells.

Apart from SmIg, the MRBC (mouse) and HLA-DR markers have been found useful in diagnosis of B cell neoplasms. Our findings confirm that the most prominent binding of mouse red cells is seen in CLL (Catovsky *et al.* 1979 and Koziner *et al.* 1977). Catovsky *et al.* (1979) report higher proportions of cells binding to mouse cells in B CLL than we have found. This probably reflects the use of neuraminidase pretreatment of cells before rosetting which has been reported as selectively increasing mouse rosettes in CLL (Catovsky *et al.*, 1976). Mouse rosette binding has been reported not to be a feature of prolymphocytic leukaemia (Catovsky *et al.*, 1979). However, Koziner *et al.* (1980) reported four cases with the features of prolymphocytic leukaemia whose neoplastic cells did bind mouse red cells. In the current study mouse rosetting was not found in the four cases of B-prolymphocytic leukaemia studied. The spectrum of patients studied by us with ML-BCLL probably includes a higher proportion of patients with low lymphocyte count than most previous studies which tend to concentrate on classical CLL with blood lymphocyte counts $>10\times10^9 l^{-1}$. We were unable to identify any feature using the marker range reported in this study which distinguished the phenotype of low and high blood lymphocyte count ML-BCLL. The HLA-DR marker is useful in that B cells at the late differentiation stage, including lymphoplasmacytoid cells, are DR negative whereas cells at the B cell stage, whether pre- or post-antigen stimulation, are DR positive. Peanut agglutinin has been reported to bind to germinal centre cells (Rose *et al.*, 1981). However, this has not proved in our hands to be a useful marker for B cell leukaemia associated with FCC tumours. This finding supports the concept that leukaemic B cells in this disease, in many cases, represent a distinct maturation phase from the tumour cells found in secondary lymphoid organs. It is arguable whether they are a precursor or a derivative of the follicle centre cells. Positive results for cells bearing PNA receptors have been observed in acute lymphoblastic leukaemia (Levin *et al.*, 1980) and for a blood cell subset which lacks HLA (Ballet, 1980). PNA has also been reported to be a marker for normal and leukaemic cells of the monocyte lineage (O'Keefe and Ashman, 1982).

Total lymphopenia as well as B cell lymphopenia was found in a significant proportion of patients in most of the different histopathological groups of B cell lymphoma. This was most obvious in patients with FCC lymphoma or high grade lymphoma where one-third and over half of the respective cases had B cell lymphopenia at presentation. The significance of this lymphopenia in B cell neoplasia is not known but it has been noted by other investigators (Burns *et al.*, 1979; Dillman *et al.*, 1981). It is sufficiently common to be a diagnostic pointer to non-Hodgkin's lymphoma. However, it is also associated with other disorders such as primary immunodeficiency, which may present with features resembling lymphoma.

The authors wish to thank their many colleagues in the West Midlands Health region who have assisted in this study. A. Cader is an Leukaemia Research Fund training fellow. P. Richardson is supported by the West Midlands Regional Health Authority research fund.

### References

BALLET, J.J. (1980). Reactivity of human lymphoid and lymphoblastoid cells with PNA: detection of a blood cell subset which lacks detectable membrane HLA. *Scand. J. Immunol.*, **11**, 555.

BENTWICH, Z., DOUGLAS, S.D., SKUTELSK, E. & KUNKEL, H.G. (1973). Sheep red cell binding to human lymphocytes treated with neuraminidase. Enhancement of T cell binding and identification of a sub-population of B cells. *J. Exp. Med.*, **137**, 1532.

BEVERLEY, P.C.L. & CALLARD, R.E. (1981). Distinctive functional characteristics of human "T" lymphocytes defined by E rosetting and a monoclonal anti T cell antibody. *Eur. J. Immunol.*, **11**, 329.

BURNS, G.F., WORMAN, C.P., ROBERTS, B.E., ROPER, C.G.L., BARKER, C.R. & CAWLEY, J.C. (1979). Terminal B cell development as seen in different human myelomas and related disorders. *Clin. Exp. Immunol.*, **35**, 180.

CATOVSKY, D., CHERCHI, M., OKOS, A., HEDGE, J. & GALTON, D.A.G. (1976). Mouse red rosettes in B lymphoproliferative disorders. *Br. J. Haematol.*, **33**, 173.

CATOVSKY, D., PITTMAN, S., O'BRIEN, M. & 8 others. (1979). Multiparameter studies in lymphoid leukaemias. *Am. J. Clin. Path.*, **72**, No. 4 suppl. p. 736.

COUTINO, A., FORNI, L. & BERNABÉ, R.R. (1980). The polyclonal expression of immunoglobulin variable region determinants on the membrane of B cells and their precursors. *Springer, Int. Sem. Immunopath.*, **13**, 171.

DHALIWAL, H.S., LING, N.R., BISHOP, S. & CHAPEL, H. (1978). Expression of immunoglobulin G on blood lymphocytes in chronic lymphocytic leukaemia. *Clin. Exp. Immunol.*, **31**, 226.

DILLMAN, R.O., ROYSTON, I., MESERVE, B.L. & GRIFFITHS, J.C. (1981). Alterations of peripheral blood B lymphocyte populations in plasma cell disorders. *Cancer*, **48**, 2211.

GAJL-PECZALSKA, K.J., BLOOMFIELD, C.D., COCCIA, P.F., SOSIN, H., BRUNNING, R.D. & KERSEY, J.H. (1975). B and T cell lymphomas. Analysis of blood and lymph nodes in 87 patients. *Am. J. Med.*, **59**, 674.

GALTON, D.A.G., CATOVSKY, D. & WILTSHAW, E. (1978). Clinical spectrum of lymphoproliferative disease. *Cancer*, **42**, 901.

GALTON, D.A.G. & MACLENNAN, I.C.M. (1982). Clinical patterns in B lymphoid malignancy. *Clinics in Haematology*, **11**, 561.

GARRETT, J.V., SCARFFE, J.H. & NEWTON, R.K. (1979). Abnormal peripheral blood lymphocytes and bone marrow infiltration in non-Hodgkin's lymphoma. *Brit. J. Haematol.*, **42**, 41.

HOWARD, J.C. (1972). The life span of recirculating and marrow-derived small lymphocytes from rat thoracic duct. *J. Exp. Med.*, **135**, 185.

JOHNSTONE, A.P. (1982). Chronic lymphocytic leukaemia and its relationship to normal B lymphopoiesis. *Immunology Today*, **3**, 343.

KAPLAN, M.E. & CLARK, C. (1974). An improved rosetting assay for detection of human T lymphocytes. *J. Immunological Methods*, **5**, 131.

KLAUS, G.G.B., HUMPHREY, J.H., KUNKL, A. & DONGWORTH, D.W. (1980). The follicular dendritic cell; its role in antigen presentation and in the generation of immunological memory. *Immunol. Rev.*, **53**, 3.

KOZINER, B., FILIPPA, D.A., MERTELSMANN, R. & 4 others. (1977). Characterisation of malignant lymphomas in leukaemic phase by multiple differentiation markers of mononuclear cells. Correlation with clinical features and conventional morphology. *Am. J. Med.*, **63**, 556.

KOZINER, B., KEMPIN, S., PASSE, S., GEE, T., GOOD, R.A. & CLARKSON, B.D. (1980). Characterisation of B-cell leukaemias: a tentative immunomorphological scheme. *Blood*, **56**, 815.

KUBAGAWA, H., VOGLER, L.B., LAWTON, A.R. & COOPER, M.D. (1980). The extent of clonal involvement in multiple myeloma. In: *Progress in Myeloma*. (Ed. M. Potter). p. 195.

LENNERT, K. (1978). *Malignant lymphomas*. Springer-Verlag, Berlin.

LEONARD, R.C.F., MACLENNAN, I.C.M., SMART, Y., VANHEGAN, R.I. & CUZICK, J. (1979). Light chain-isotope associated suppression of normal plasma cell numbers in patients with multiple myeloma. *Int. J. Cancer*, **24**, 385.

LEVIN, S., RUSSELL, E.C., BLANCHARD, D., MCWILLIAMS, N.B., MAURER, H.M. & MOHANAKUMAR, T. (1980). Receptors for peanut agglutinin in childhood acute lymphoblastic leukaemia: possible clinical significance. *Blood*, **55**, 37.

LING, N.R. & MACLENNAN, I.C.M. (1981). Analysis of lymphocytes in blood and tissues. In: *Techniques in Clinical Immunology*. (Ed. R.A. Thompson). 2nd ed. p. 222. Blackwell Scientific Publications.

LOWE, J., HARDIE, D., JEFFERIS, R. & 8 others (1981). Properties of monoclonal antibodies to human immunoglobulin kappa and lambda chains. *Immunology*, **42**, 649.

MELLSTEDT, H., PETTERSSON, D. & HOLM, G. (1982). Idiotype-bearing lymphoid cells in plasma cell neoplasia. *Clin. Haematol.*, **11**, 65.

O'KEEFE, D. & ASHMAN, L. (1982). Peanut agglutinin: a marker for normal and leukaemic cells of the monocyte lineage. *Clin. Exp. Immunol.*, **48**, 329.

PETTERSSON, D., MELLSTEDT, H. & HOLM, G. (1979). Immunoglobulin isotypes on monoclonal blood lymphocytes in human plasma cell myeloma. *J. Clin. Lab. Immunol.*, **3**, 93.

PETTERSSON, D., MELLSTEDT, H. & HOLM, G. (1980). Monoclonal B lymphocytes in multiple myeloma. *Scand. J. Immunol.*, **12**, 375.

PREUD'HOMME, J.-L., KLEIN, M., LABAUME, S. & SELIGMANN, M. (1977). Idiotype-bearing and antigen-binding receptors produced by blood T lymphocytes in a case of human myeloma. *Eur. J. Immunol.*, **7**, 840.

ROSE, M.L., HABESHAW, J.A., KENNEDY, R., SLOANE, J., WILTSHAW, E. & DAVIES, A.J.S. (1981). Binding of peanut lectin to germinal centre cells: a marker for B cell subsets of follicular lymphoma. *Brit. J. Cancer*, **44**, 68.

RUBINSTEIN, L.J., YEH, M. & BONA, C.A. (1982). Idiotype-anti-idiotype network. II. Activation of silent clones by treatment at birth with idiotypes is associated with the expansion of idiotype-specific helper T cells. *J. Exp. Med.*, **156**, 506.

STATHOPOULOS, G. & ELLIOTT, E.V. (1974). Formation of mouse or sheep blood cell rosettes by lymphocytes from normal and leukaemic individuals. *Lancet*, **i**, 600.

SWERDLOW, S.H., DHALIWAL, H.S., MURRAY, L., STANSFELD, A.G. & HABESHAW, J. (1983). Centrocytic lymphoma: A distinct clinicopathologic and immunologic entity. *J. Clin. Invest.*, **48**, 83A.